# Predicting Magnetoelectric Coupling in Layered and Graded Composites

**DOI:** 10.3390/s17071651

**Published:** 2017-07-19

**Authors:** Mirza Bichurin, Vladimir Petrov, Alexander Tatarenko

**Affiliations:** Institute of Electronic and Information Systems, Novgorod State University, Veiky Novgorod 173003, Russia; vladimir.petrov@novsu.ru (V.P.); alexandr.tatarenko@novsu.ru (A.T.)

**Keywords:** magnetoelectric effect, magnetic field induced ME effect, graded magnetostrictive material, composites, graded piezoelectric, multiferroic, bimorph, bending resonance, nomograph method

## Abstract

Magnetoelectric (ME) interaction in magnetostrictive-piezoelectric multiferroic structures consists in inducing the electric field across the structure in an applied magnetic field and is a product property of magnetostriction and piezoelectricity in components. ME voltage coefficient that is the ratio of induced electric field to applied magnetic field is the key parameter of ME coupling strength. It has been known that the ME coupling strength is dictated by the product of the piezoelectric and piezomagnetic coefficients of initial phases. As a result, using the laminates with graded piezoelectric and piezomagnetic parameters are a new pathway to the increase in the ME coupling strength. Recently developed models predict stronger ME interactions in composites based on graded components compared to homogeneous ones. We discuss predicting the ME coupling strength for layered structures of homogeneous and compositionally graded magnetostrictive and piezoelectric components based on the graphs of ME voltage coefficients against composite parameters. For obtaining the graphs, we developed equations for ME output in applied magnetic field for possible modes of operation and layered structure configurations. In particular, our studies have been performed on low-frequency ME coupling, enhanced ME effect in electromechanical resonance (EMR) region for longitudinal and bending modes. Additionally, ME coupling at magnetic resonance in magnetostrictive component and at overlapping the EMR and magnetic resonance is investigated. We considered symmetric trilayers and asymmetric bilayers of magnetostrictive and piezoelectric components and multilayered structures based on compositionally stepped initial components.

## 1. Introduction

Magnetoelectric (ME) interaction in magnetostrictive-piezoelectric multiferroic structures consists in inducing the electric (magnetic) field in an applied magnetic (electric) field and is a product property of magnetostriction and piezoelectricity in components. The coupling between the magnetostrictive and piezoelectric subsystems is implemented by mechanical strains. The electric to external magnetic field ratio referred to as ME voltage coefficient is the key parameter of ME coupling strength. Ferromagnetic-ferroelectric composites are under intense study in recent years for potential use in sensors, energy harvesters, and for signal processing. Composites of magnetostrictive metals, alloys, or oxides and piezoelectric lead zirconate titanate (PZT), barium titanate, lead magnesium niobate-lead titanate (PMN-PT), or quartz-like crystals demonstrated a giant ME effect in the low frequency and electromechanical resonance (EMR) regions [1,2,3,4,5,6,7,8,9,10,11,12,13,14,15,16,17]. Using nomographs for a quick test of ME composites for applications where a rapid estimate is relevant and useful was reported recently.

The induced polarization P is expressed in terms of applied magnetic field *H* by the expression, P = αH, with α being the ME susceptibility. ME effect in Cr_2_O_3_ was first investigated. The strongest ME coupling obtained for a single crystal Cr_2_O_3_ [1] is α = 20 mV/(cm·Oe). Strong ME coupling in magnetostrictive-piezoelectric composites was recently observed due to appropriate choice of material properties and proper sample design. The most investigated composites are Ni-Co ferrite/PZT, Terfenol-D/PZT and Metglas/PZT. The highest ME voltage coefficient of 500 V/cm·Oe was found for an amorphous magnetostrictive alloy/piezofiber layered structure [3]. These studies enabled making ME structures with high ME coefficients in a wide frequency region and practical application possibility [4,5,18].

A theoretical model of the anisotropic ME effect as a function of the crystallographic orientation in trilayers of Metglas and piezoelectric single crystals was discussed recently [19,20]. The peak value of direct transversal ME voltage coefficients was estimated. The magnetoelectric coupling can be increased (up to 500 V/cm·Oe) by using the appropriate cut angle of LiNbO_3_, thickness of the LiNbO_3_ and Metglas layers, and a better bonding between ferromagnetic and ferroelectric layers [21]. Multiferroic bulk composites and nanocomposites were considered in respect to theoretical phenomenological and first-principles estimates of ME effect and their applications [22].

Piezoelectric component of a layered structure must be polarized to reveal high piezoelectric coupling coefficient and strong ME coupling. The poling procedure includes heating the sample to 450 K, and cooling to room temperature in applied dc electric field of E = 20–50 kV/cm. A laminate under study is placed into the bias magnetic field up to 18 kOe produced by an electromagnet. Helmholtz coils generate ac magnetic field *δH* applied to the magnetostrictive layer parallel to bias field *H*. The ac voltage *δV* measured across the piezoelectric layer is used for calculating the induced electric field *δE*. The ME coefficient *α_E_* is estimated for three orientations of magnetic and electric fields: (1) *α_E_*_,31_ is measured for transverse fields’ orientation which involves *H* and *δH* parallel to the sample plane and *δE* perpendicular to the sample plane, (2) *α_E_*_,33_ is estimated for longitudinal fields’ orientation with all the three fields perpendicular to sample plane, and (3) *α_E_*_,11_ is defined as ME voltage coefficient for in-plane fields’ orientation with in-plane poling direction, electric and magnetic fields. When the laminate undergoes electromechanical resonance (EMR), a significant increase in ME coupling strength is obtained [23,24,25]. This phenomenon is of vital importance for practical application. A summary of the most recent advances in the physics and applications of the magnetoelectric effect in composite multiferroics was recently published [26].

Other phenomenon of technological importance is ME effect at FMR. A dc electric field generates a strain of the piezoelectric phase which is transferred to magnetic phase. Stressed magnetic layer shows a shift in the FMR frequency. In what follows we estimate ME effects in the wide frequency range.

Because the ME coupling in magnetostrictive-piezoelectric composites is a result of mechanical strains, the key parameters that determine the ME coupling strength are the piezoelectric coefficient *d* and the piezomagnetic coefficient *q*. The ME coupling strength can be increased by grading these parameters. For example, using the layered structure with graded piezoelectric or/and piezomagnetic layer in out-of-plane direction gives rise to an additional bending moment. The bending moments are known to influence the ME effect strength. Theoretical models that considered ME coupling in the low-frequency and EMR regions in bilayers with out-of-plane grading of *d* and *q* were described recently. Estimates of ME voltage coefficient showed that the ME interaction in graded structures is 50–60% stronger compared to homogeneous systems [14,15]. In addition, it was shown that internal magnetic and electric fields in graded materials enable one to simplify the ME material based devices design since it is possible to exclude elements creating external bias field, and simplify the technological process of material production since it is possible to exclude preliminary polarization of material sample [16].

This work deals with ME interactions in layered magnetostrictive-piezoelectric composites based on homogeneous and functionally graded components. In an asymmetric ferromagnetic-piezoelectric bilayer, an ac magnetic field produces axial and flexural strains simultaneously [11,17]. The asymmetry related flexural deformation can be produced in functionally stepped ferromagnetic or/and piezoelectric layer [15,18].

## 2. Low-Frequency Magnetoelectric Coupling

Here we present a detailed discussion of low-frequency and resonance ME effects in magnetostrictive-piezoelectric bilayers and trilayers including structures of homogeneous initial layers and structures of piezoelectric with stepped *d* and ferromagnetic layer. The step in *d* is accomplished with the use of two piezoelectric layers with opposite poling directions.

### 2.1. Magnetoelectric Coupling in Multilayers of Homogeneous Piezoelectric and Magnetostrictive Components

The transverse fields’ orientation that corresponds to out-of-plane *E* and *δE* and in-plane *H* and *δH* is discussed here since this orientation provides the minimal demagnetizing fields and related decrease in ME interaction. The transverse ME voltage coefficient is determined in terms material parameters of initial components:

(1) αE,31=E3H1=  −V(1−V)(q11m+q21m)d31pε33p(s12m+s11m)v+ε33p(s11P+s12P)(1−V)−2d312p(1−V)  

The expression for transverse ME voltage coefficient of magnetostrictive-piezoelectric trilayer can be reduced to the following 1-D approximation:

(2) αE,31=V(1−V)xε0[s11mV+s11P(1−V)]

where x=q11md31pε33pε0, *^p^s*_11_, *^m^s*_11_, *^p^d*_31_, *^p^ε*_33_ and ^m^*q*_11_ are compliance of piezoelectric layer, compliance of piezomagnetic layer, piezoelectric coupling coefficient and permittivity of piezoelectric layer, piezomagnetic coupling coefficients of piezomagnetic phase. For simplicity, we assume the electromechanical coupling coefficient to be small compared to unity: K312P=d312ps11pε33p<<1. 

**Figure 1:**
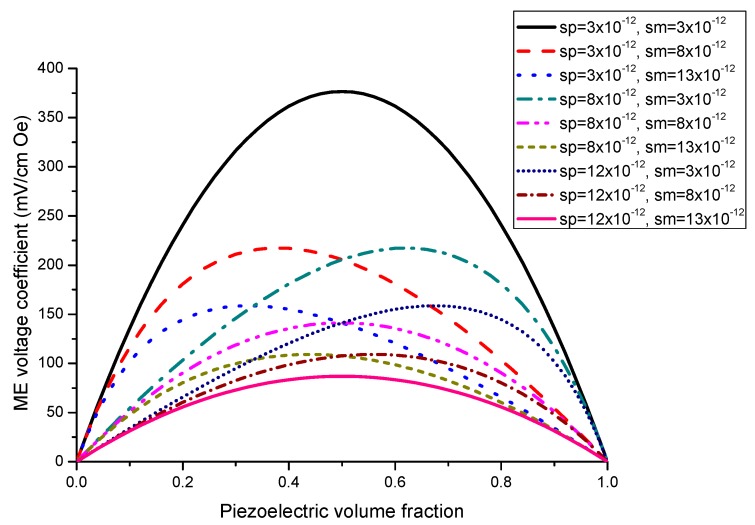
Piezoelectric volume fraction dependence of transverse ME voltage coefficient for symmetric laminate of homogeneous magnetostrictive and piezoelectric layers with different values of component compliances for *x* = 0.5 × 10^−22^ (in SI units) [27].

**Figure 2:**
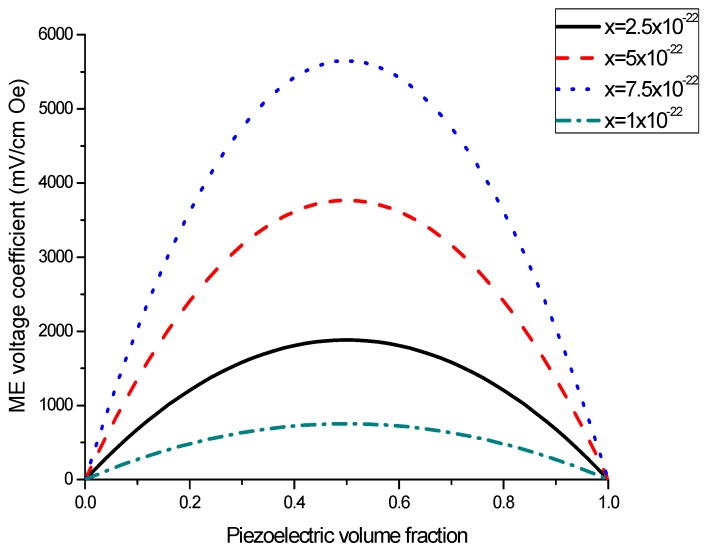
Piezoelectric volume fraction dependence of transverse ME voltage coefficient for symmetric layered structure of magnetostrictive and piezoelectric components for different *x*-values [27].

The nomograph method described here is supposed to enable estimating the ME voltage coefficients from given material parameters of initial components [27]. Figure 1 and Figure 2 show the piezoelectric volume fraction dependence of ME voltage coefficients. This dependence is exemplified by transverse ME voltage coefficient. Transverse fields’ orientation is known to provide minimal demagnetizing fields.

Calculation of ME voltage coefficient for the nonsymmetric structure takes into consideration the bending strain. For this case, we get following equation for ME voltage coefficient:

(3) δE3δH1=[1s11p+s11mr3]q11md31pε33ps11p[2rs11m(2+3r+2r2)+s11p]+s112mr4

where *r* = *^p^t/^m^t* with *^p^t* and *^m^t* being the thickness of initial piezoelectric and magnetostrictive layer, respectively.

In deriving Equation (3), we used a short-cut computation assuming K312p<<1 similarly to when deriving Equation (2).

**Figure 3:**
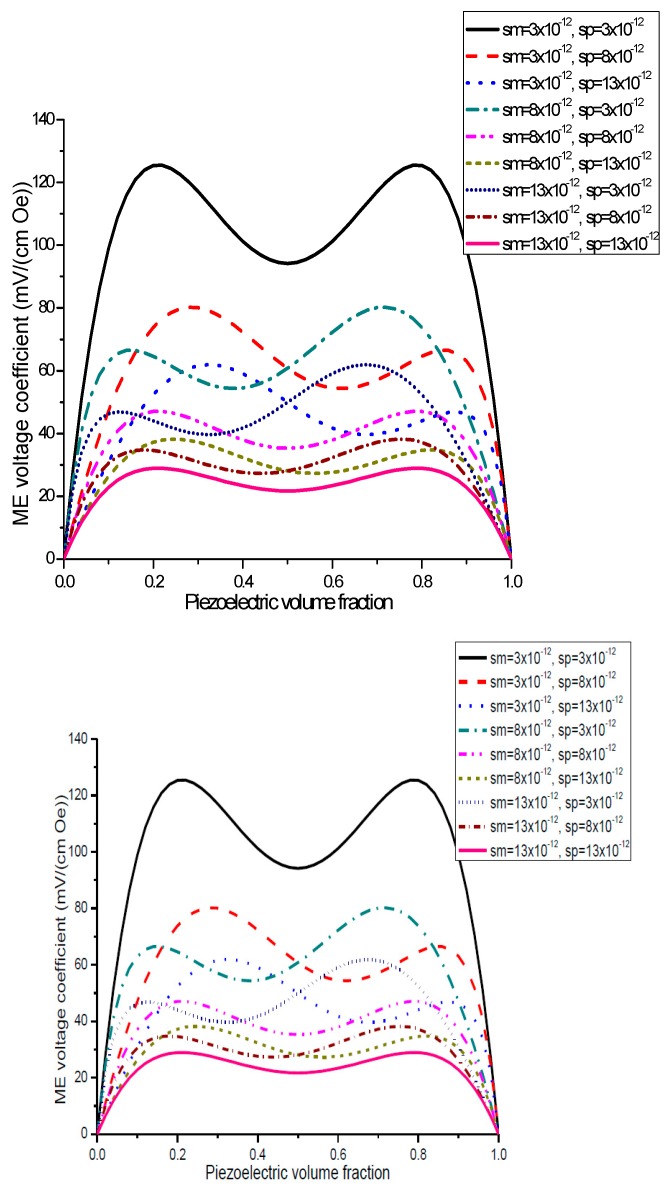
Plots of transverse ME voltage coefficient vs. piezoelectric volume fraction for magnetostrictive -piezoelectric bilayer with several compliances for *x* = 0.5 × 10^−22^ (in SI units) [27].

**Figure 4:**
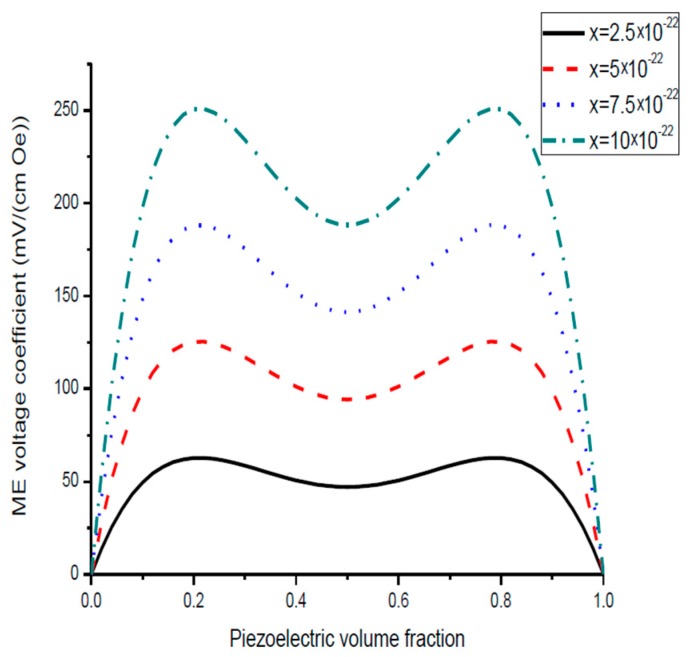
Plots of transverse ME voltage coefficient vs. piezoelectric volume fraction for magnetostrictive—piezoelectric bilayer at different *x* values in SI units [27].

Plots of ME voltage coefficient vs. piezoelectric volume fraction are shown in Figure 3 and Figure 4 for different values of compliance and product of piezoelectric and piezomagnetic coefficient.

### 2.2. Low-Frequency Magnetoelectric Effect in Trilayer of Stepped Piezoelectric Layer and Magnetostrictive Component

Two PZT layers are assumed to be polarized in opposite directions to enable observing the ME interaction due to bending strain. The obtained ME coefficients for the structure based on piezoelectric bimorph is found to be two times higher compared to the traditional bilayer of PZT and Metglas [15,18].

To get the explicit expression for ME voltage coefficient, we consider a laminate of piezoelectric bimorph and magnetostrictive layer. The poling direction for first piezoelectric layer is opposite to that for second piezoelectric layer. The sample is supposed to be a thin plate with thickness *t* that is small compared to width *w*. The sample length *L* is assumed to be much greater than width *w*. The poling direction is parallel to thickness direction *Z*. The dc bias and ac magnetic fields are applied parallel to length direction. In this case, the stress tensor has only one nonzero component, *X*-components. The piezomagnetic coupling produced the strain of the magnetostrictive layer. Because the stresses in both layers are not applied centrally, the bending moment with respect to *Y*-axis appears due to the asymmetry of the sample. Thus, flexural deformations are produced. To take into account the bending strain, the axial strains of each layer are considered as linear functions of *Z*. 

The theoretical modeling is based on the well-known elasticity equations, material equations for piezoelectric and magnetostrictive layers:

(4) *^p^*^1^*S*_1_ = *^p^s*_11_*^p^*^1^*T*_1_* + ^p^d*_31_*^p^*^1^*E*_3_; *^p^*^1^*D*_3_ = *^p^d*_31_*^p^*^1^*T*_1_*+ ^p^ε*_33_*^p^*^1^*E*_3_; *^p^*^2^*S*_1_ = *^p^s*_11_*^p^*^2^*T_j_ − ^p^d*_31_*^p^E*_3_; *^p^*^2^*D*_3_ = *−^p^d*_31_*^p^*^2^*T_i_ + ^p^ε*_33_*^p^*^2^*E*_3_; *^m^S*_1_ = *^m^s*_11_*^m^T*_1_*+ ^m^g*_11_*^m^B*_1_*;* *^m^H*_1_ = *−^m^g*_11_*^m^T_i_ + 1/^m^μ*_11_*^m^B*_1_;

where *S*_1_ and *T*_1_ are *X*-components of strain and stress, *E*_3_ and *D*_3_ are electric field and electric induction, *H*_1_ and *B*_1_ are magnetic field and magnetic induction, *d*_31_ and *g*_11_ are piezoelectric and piezomagnetic coefficients, *^p^s*_11_ and *^m^s*_11_ are elastic compliance coefficients, *ε*_33_ is the permittivity, and *μ*_11_ is permeability. The inferior characters *p*_1_, *p*_2_, and *m* denote the piezoelectric layers and ferromagnetic layer, correspondingly. Calculation is analogous to recently discussed investigation of ME effect in a layered gradient system [14]. To take into account the cylindrical bending of trilayer, the axial strains of each layer are assumed to be varying along the thickness direction: *^p^*^1^*S*_1_ = *^p^*^1^*S*_10_ *+ z_p_*_1_*/R*; *^p^*^2^*S*_1_ = *^p^*^2^*S*_10_ *+ z_p_*_2_*/R*; *^m^S*_1_ = *^m^S*_10_ *+ z_m_/R* where *^i^S*_10_ are the central deformations along *x*-axis at *z_i_* = *0*, *R* is the radius of bend, and *z_i_* is reckoned from central plane of *i*-layer. The central strains are related by the equations: *^p^*^2^*S*_10_ *– ^p^*^1^*S*_10_ = *h*_2_*/R*_1_, *^m^S*_10_ *− ^p^*^1^*S*_10_ = *h*_1_*/R*_1_ where *h*_1_ = *(^p^*^1^*t + ^m^t)/2* and *h*_2_ = *(^p^*^1^*t + ^p^*^2^*t)/*2 with *^p^*^1^*t*, *^p^*^2^*t*, and *^m^t* being thicknesses of layers. For equilibrium condition, the axial forces and the rotating moments should be balanced:

(5) $${}^{p1}F_{1}{+}^{p2}F_{1}{+}^{m}F_{1}=0,$$

(6) p1F1h1+p2F1(h1+h2)=M1m+M1p1+M1p2,

where Fi1=∫−ti/2it/2iT1dz1, M1i=∫−ti/2it/2ziiT1dzi.

Solving Equations (4)–(6) provides the stress components. Substituting the value of stress components into the open circuit condition enables one to derive an analytical expression for ME voltage coefficient: αE 31=−d31pt H1ε33p(∫−tp12tp12(Tp11+Tp21)dz) with *H*_1_ denoting the applied ac magnetic field. This expression can be reduced to a simplified one for equal thickness of PZT layers and the electromechanical coupling factor squared negligibly small compared to unity.

(7) αE 31=3q11ms11m(1−v)v3d31p2{(s11m−s11P)v[(s11m−s11P)v3+4s11P(v2+1)−6s11Pv]+s112P}ε33p

where piezoelectric volume fraction is defined as *v* = *(^p^*^1^*t + ^p^*^2^*t)/t*.

**Figure 5:**
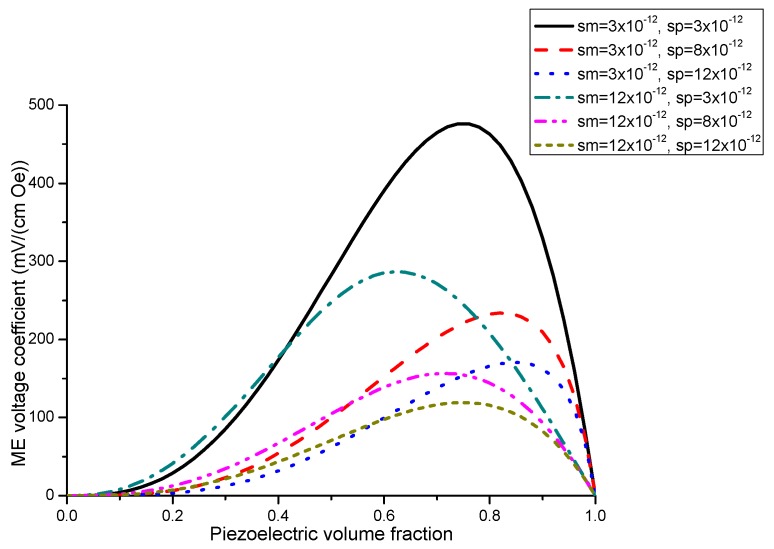
Dependence of transverse ME voltage coefficient on piezoelectric volume fraction for layered structure of stepped piezoelectric and magnetostrictive component for different compliances for x=q11md31pεp33ε0=1×10−22 (in SI units).

**Figure 6:**
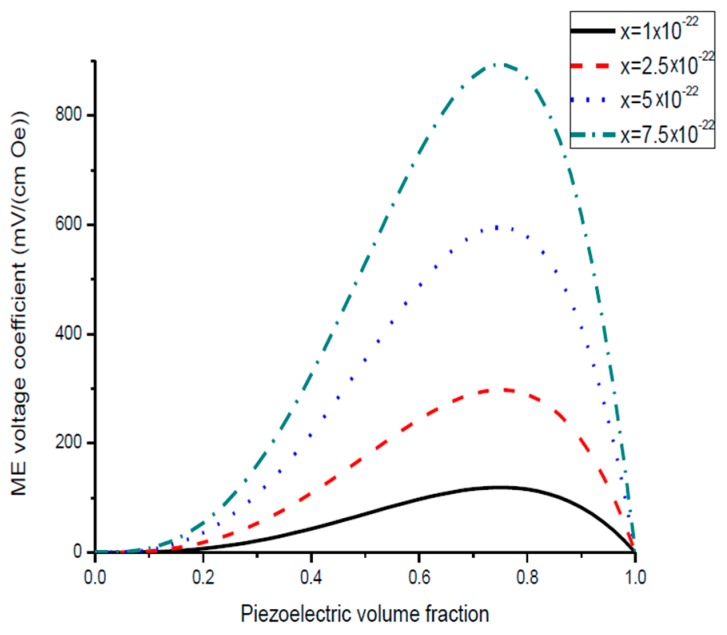
Dependence of transverse ME voltage coefficient on piezoelectric volume fraction for layered structure of stepped piezoelectric and magnetostrictive component at different *x* values in SI units.

**Figure 7:**
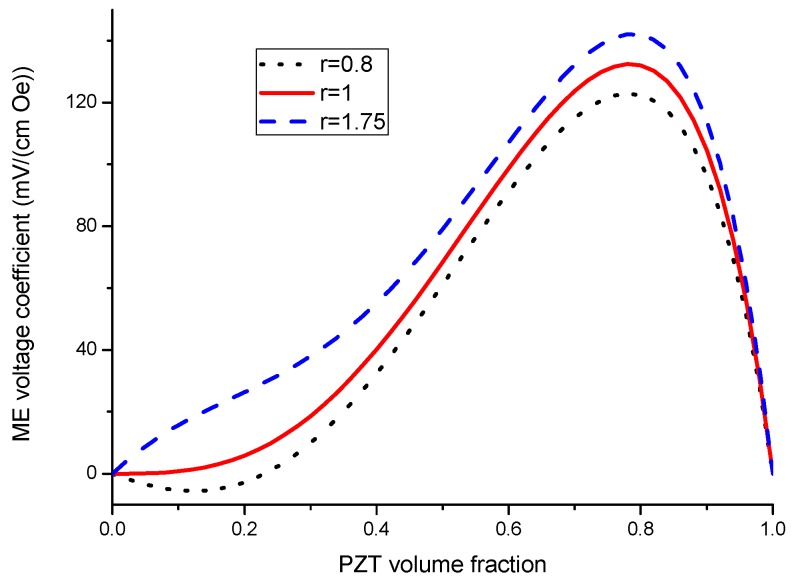
Dependence of ME voltage coefficient on piezoelectric volume fraction for layered structure of stepped piezoelectric and magnetostrictive material at different thickness ratio *r* = *^p^*^1^*t/^p^*^2^*t* (*^p^*^1^*t* and *^p^*^2^*t* are the thicknesses of two piezoelectric layers) [28]*.*

Equation (7) reveals that ME voltage coefficient is principally specified by piezoelectric and piezomagnetic coefficients of initial phases and their volume fraction. Thus, one can utilize the most appropriate piezoelectric and magnetostrictive phases and optimal thickness of the components to observe an increase in the ME coupling strength for the laminate of stepped piezoelectric and magnetostrictive component. Figure 5 and Figure 6 show the plots of ME voltage coefficient vs. piezoelectric volume fraction for layered structure of stepped piezoelectric and magnetostrictive layer for several values of compliance and product of piezoelectric and piezomagnetic coefficient. Estimated dependence of ME voltage coefficient on piezoelectric volume fraction for layered structure of stepped piezoelectric and magnetostrictive layer is shown in Figure 7 for different thickness ratio *r* = *^p^*^1^*t/^p^*^2^*t* (*^p^*^1^*t* and *^p^*^2^*t* are the thicknesses of two piezoelectric layer).

## 3. Magnetoelectric Coupling at Bending Mode

### 3.1. Magnetoelectric Coupling at Bending Mode in Layered Structures of Homogeneous Components

In what follows, we discuss ME coupling in the bending mode region of a bilayer with single-sided support. The bilayer deflection should satisfy the equation of flexural vibrations discussed in our models in Ref. [11]. To solve these equations, we used the necessary boundary conditions for solving the equation of motion: the out-of-plane displacement and its derivative become zero at clamped end and rotational moment and transverse force become zero at free end. Assuming K312p<<1 and K112m<<1 (mK112=qm112sm11μm11) with *^m^µ*_11_ denoting the absolute permeability of magnetic layer) results in the equation for EMR frequency *cosh(kL)∙cos(kL)* = *−*1 with *k* being the wave number.

The ME voltage coefficient at bending mode frequency can be estimated as

**Figure 8:**
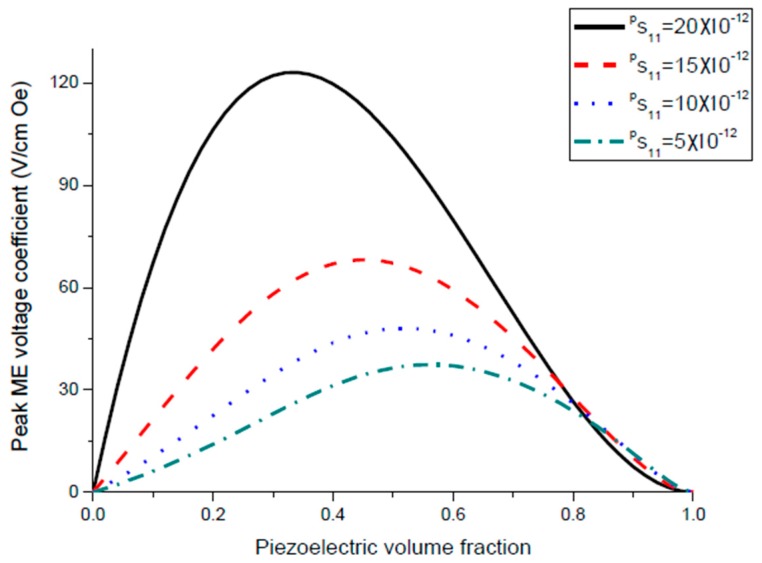
Variation of peak ME voltage coefficient with piezoelectric volume fraction at bending mode of magnetostrictive-piezoelectric bilayer for *^m^s*_11_ = 5 × 10^−12^ m^2^/N and different values of *^p^s*_11_. Value of *x*_1_ is equal to 5 × 10^−20^ (in SI units) [27].

**Figure 9:**
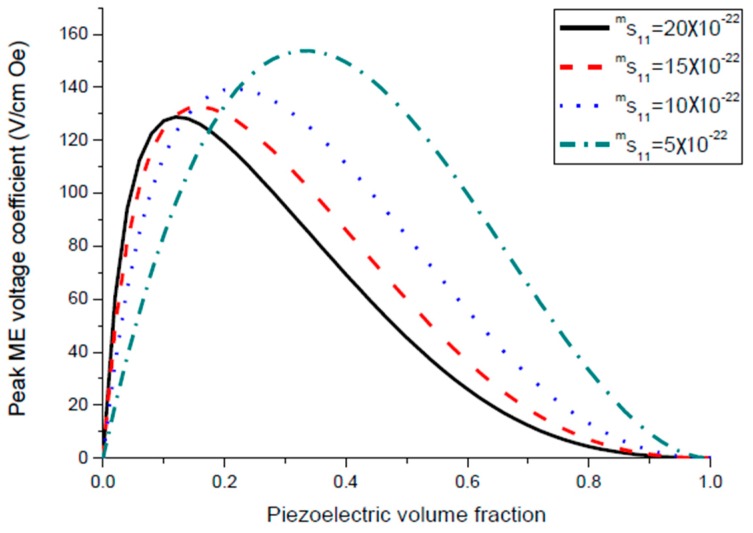
Variation of peak ME voltage coefficient with piezoelectric volume fraction at bending mode of magnetostrictive-piezoelectric bilayer for *^p^s*_11_ = 5 × 10^−12^ m^2^/N and different values of *^m^s*_11_. Value of *x*_1_ is equal to 5 × 10^−20^ (in SI units).

(8) δE3δH1=0.0766Qbtm(tm+2z0)(2z0−tp)d31pq11mDs11ps11mε33p

where *D* and *Q_a_* are bending rigidity of the sample and the Q-value for the bending mode. The maximum value of ME voltage coefficient is principally specified by Q value, piezoelectric and piezomagnetic coupling coefficients, and thickness of initial components. Equation (8) can be written in a more adequate form αE,  31=0.0766tm(tm+2z0)(2z0−tp)x1Ds11ps11mε0 with x1=d31pq11mQbε33pε0. Variation of peak ME voltage coefficient at bending mode of magnetostrictive-piezoelectric bilayer with piezoelectric volume fraction is shown in Figure 8 and Figure 9 for different values of the piezoelectric and magnetostrictive phase compliances.

**Figure 10:**
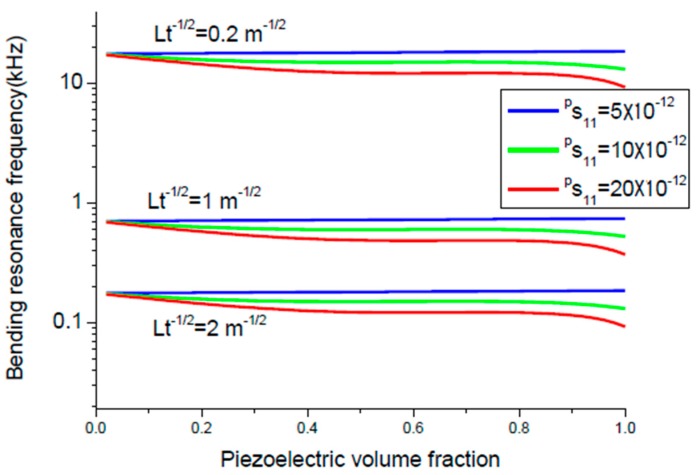
Variation of bending resonance frequency of magnetostrictive-piezoelectric bilayer with volume fraction of piezoelectric component for *^m^s*_11_ = 5 × 10^−12^ m^2^/N and particular *^p^s*_11_ and *Lt^−^*^½^ [27].

**Figure 11:**
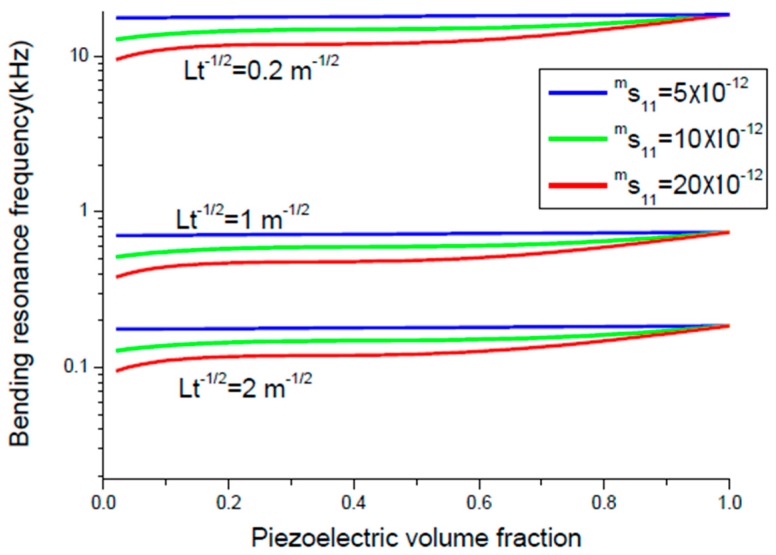
Variation of bending resonance frequency of magnetostrictive-piezoelectric bilayer with volume fraction of piezoelectric component for *^p^s*_11_ = 5 × 10^−12^ m^2^/N and particular *^m^s*_11_ and *Lt^−^*^½^ [27].

The flexural resonance frequency is specified by equation fr=1.758πL2Dρptp+ρmtm and is determined by elastic compliances, thickness of initial components, and ratio $\frac{L}{\sqrt{t}}$. Variation of flexural resonance frequency of magnetostrictive-piezoelectric bilayer with volume fraction of piezoelectric component is presented in Figure 10 and Figure 11 for particular compliance of piezoelectric and magnetostrictive layers.

### 3.2. Magnetoelectric Coupling at Bending Mode in Layered Structures of Stepped Components

The theory of enhanced ME interactions at electromechanical resonance in graded ME composites was developed recently [15]. In the study, a technique was developed for estimates of ME coefficients at the bending resonance frequency in a bilayer of graded piezoelectric or/and ferromagnetic layers with linearly varying piezoelectric or/and piezomagnetic coefficients. Our focus here is modeling ME effects in bending resonance region in composites with stepped piezoelectric coefficients.

For a laminate with thickness small compared to width and width small in comparison to length, the equation of flexural vibrations has the form [14]:

(9) $$\nabla^{2}\nabla^{2}w+\frac{\rho \;t}{D}\frac{\partial^{2}w}{\partial t^{2}}=0$$

where ∇^2^∇^2^ is biharmonic operator, *t* and *ρ* are thickness and density’s mean value with respect to sample volume, *w* is the out-of-plane displacement, and *D* is flexural rigidity of laminate. For a trilayer of magnetic and two piezoelectric layers, *t* = *^m^t + ^p^*^1^*t + ^p^*^2^*t*, *ρ* = *(^m^ρ^m^t + ^p^*^1^*ρ^p^*^1^*t + ^p^*^2^*ρ^p^*^2^*t)/t*, where *^m^t*, *^p^*^1^*t*, and *^p^*^2^*t* are thicknesses of piezomagnetic and two piezoelectric layers, correspondingly, *^m^ρ*, *^p^*^1^*ρ*, *^p^*^2^*ρ* are densities of layers.

The arbitrary constants that enter into the general solution of Equation (8) should be found from boundary conditions at the sample ends. Next we consider a trilayer with single-sided support since this type of end fixity condition results in the lowest resonance frequency. The out-of-plane displacement (deflection) and *x*-derivative of out-of-plane displacement are known to vanish at clamped end (*x* = 0). The same is true of the moment of rotation *M*_1_ and shearing force *V*_1_ at *x* = *L*. The stress *T*_1_ which enters into the expression for moment of rotation and shearing force is expressed from Equations (1) with regard to equation: $S_{1}=-z\frac{\partial^{2}w}{\partial x^{2}}$.

Once the stresses are found from solution of Equation (8), open circuit condition ∫ADp3dx=0 (A is the area of cross section) enables us to obtain the ME voltage coefficient:

(10) αE 31=YmYpd31pq11mtm(2z0+tm)[2z0(tp2−tp1)−2tp1tp2−tp22+tP12](r1r4+r2r3)4εp33DkL(tp1+tp2+tm)(1+r1r3)

where $r_{1}=\cos(kL);r_{2}=\sin(kL);\;\;r_{3}=\cosh(kL);\;\;r_{4}=\sinh(kL);\;\;$

z0=Ymt2m−Yp1t2p1−Yp2tp2(2tp1+tp2)2(Ymtm+Yp1tp1+Yp2tp2); k4=ω2(ρmtm+ρp1tp1+ρp2tp2)D−1.

**Figure 12:**
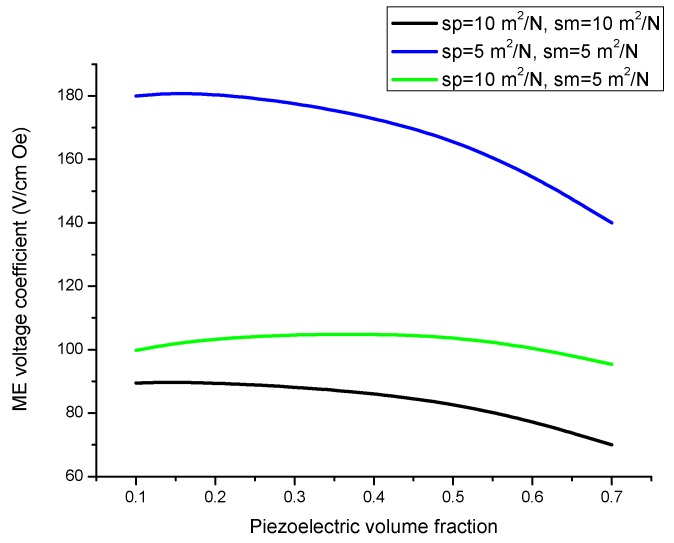
Variation of peak ME voltage coefficient with volume fraction of piezoelectric phase *x*_1_-value equals 5 × 10^−20^ (in SI units).

**Figure 13:**
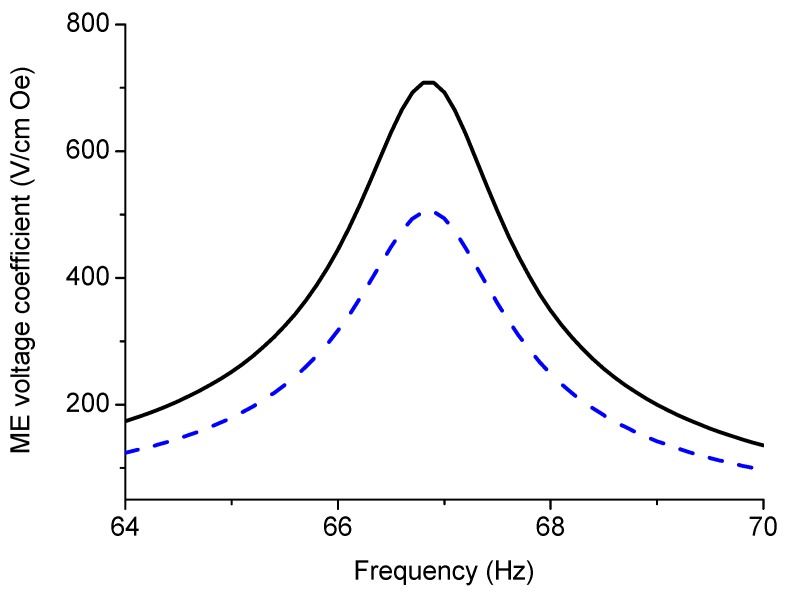
ME voltage coefficient vs. frequency in the bending mode region for layered structure of magnetostrictive layer and stepped piezoelectric component (solid line) and for piezoelectric/magnetostrictive bilayer (dot line). Thickness of first and second piezoelectric and Metglas layers equals 0.1, 0.3, and 0.2 mm, correspondingly. *x*_1_-value equals 5 × 10^−20^ (in SI units).

Equation (10) shows that the key parameters that govern ME effect in the bending mode region are the piezoelectric and piezomagnetic coefficients of initial components. A considerable increase in ME coupling strength is observed when the applied magnetic field frequency approaches the flexural resonance frequency. Neglecting the piezoelectric and piezomagnetic properties results in the following resonance condition: cos(kL)cosh(kL)=−1. This expression agrees with resonance condition for a bar free at one end and rigidly clamped at the other end. Variation of peak ME voltage coefficient with volume fraction of piezoelectric phase is presented in Figure 12 for several component compliances. Frequency dependence of ME voltage coefficient in the bending mode region for laminate of magnetostrictive layer and stepped piezoelectric component is shown in Figure 13.

## 4. Magnetoelectric Effect in the Axial Mode Region

The longitudinal vibrations of a magnetostrictive/piezoelectric laminate are governed by the equation of axial motions provided in Ref. [11]. As an example, we apply this equation to the laminate free at both ends. Assuming K112p<<1 enables one to obtain a simplified expression for the fundamental EMR frequency:

(11) f=12Ls11P+rs11ms11Ps11m(rρp+ρm)

The peak ME voltage coefficient at EMR frequency for axial mode reduces to following expression:

δE3δH1=8Qaπ2rq11md31pε33p(rs11m+s11p)(r+1)

or

(12) αEQa=8π2V(1−V)q11md31pε33p[Vs11m+(1−V)s11p]

where *Q_a_* is the Q-value for the longitudinal resonance.

**Figure 14:**
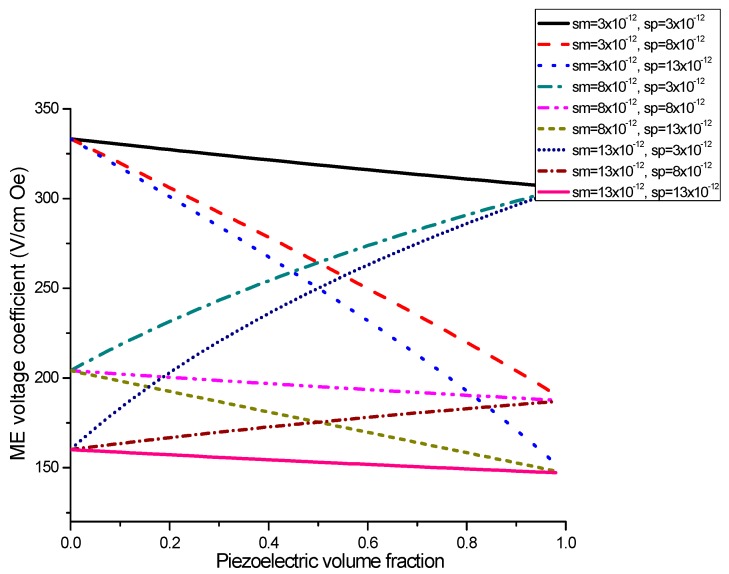
Piezoelectric volume fraction dependence of EMR frequency for longitudinal mode of magnetostrictive-piezoelectric laminate with length of 10 mm [27].

**Figure 15:**
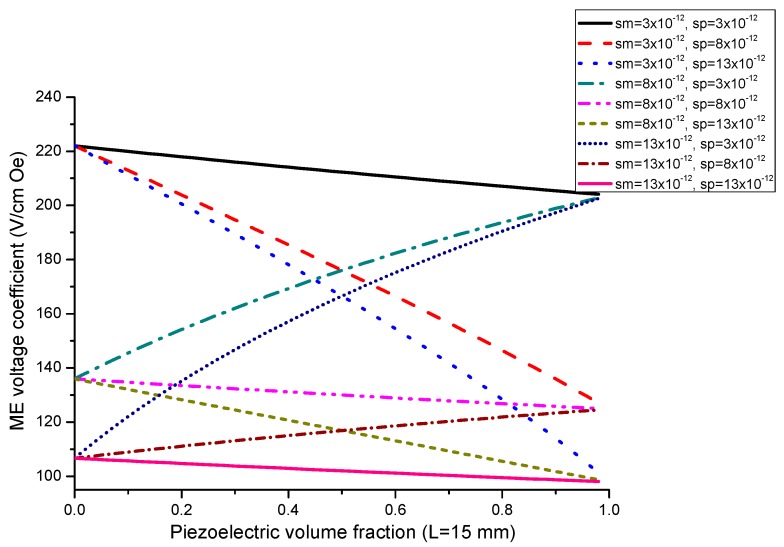
Piezoelectric volume fraction dependence of EMR frequency for longitudinal mode of magnetostrictive-piezoelectric laminate with length of 15 mm [27].

Notice that the peak ME voltage coefficient and resonance frequency are governed by Equations (11) and (12) both for bilayer and for trilayer structures of homogeneous components. It should be emphasized that the ME voltage coefficient divided by Q-value varies with piezoelectric volume fraction similarly to low-frequency ME coefficient which is determined by Equation (2). Dependence of EMR frequency on piezoelectric volume fraction is given in Figure 14 and Figure 15.

## 5. Magnetoelectric Coupling in FMR Region

Here we discuss the ME interactions in microwave region in two-layer structures based on piezoelectric and single crystal ferrite with graded anisotropy induced by dc electric field. The ferrite plate is bonded to piezoelectric layer. Electric field applied to piezoelectric layer gives rise to flexural deformations due to the asymmetry of structure. Thickness-dependent strains are transmitted to the ferrite phase and induce the magnetic anisotropy which varies across the thickness [11]. Apart from the fact that the low line width facilitates the precise measurements of ME effect, the model described here provides evidence for electric control of magnetic resonance line width. As opposed to the prediction of variation in ferromagnetic resonance absorption due to the applied electric field to graded ferroelectric, we suggest using the ferrite/piezoelectric bilayer with homogeneous piezoelectric layer. In this case, the applied electric field gives rise to flexural deformations that result in the thickness-dependent induced anisotropy and thus in the shift and broadening of FMR line. This can be useful for microwave applications. Magnetic susceptibility with regards to ME coupling was estimated in terms of component material parameters.

To estimate the magnetic anisotropy induced by applied electric field, we discuss a ferrite/piezoelectric bilayer. Ferrite is assumed to be in a state of saturation due to out-of-plane dc magnetic field. To find the magnetic susceptibility, we solve the Landau-Lifshitz equation of motion for magnetization which is nonlinear in the main case. However, the equation can be linearized provided that ac components of magnetic field and magnetization are small compared to dc components. In that case, solution can be easily found by using the effective demagnetization factor method. The magnetic susceptibility is known to be expressed as:

(13) $$\chi^{M}=\left[\begin{array}{ccc} \chi_{1} & \chi_{s}+i\chi_{a} & 0\\ \chi_{s}-i\chi_{a} & \chi_{2} & 0\\ 0 & 0 & 0 \end{array}\right],$$

Where

$$\begin{array}{l} \chi_{1}=D^{-1}\gamma^{2}M_{0}[H_{03^{\prime }}^{}+M_{0}\sum_{i}(N_{2^{\prime }2^{\prime }}{}^{i}-N_{3^{\prime }3^{\prime }}^{i})];\\ \chi_{2}=D^{-1}\gamma^{2}M_{0}[H_{03^{\prime }}^{}+M_{0}\sum_{i}(N_{1^{\prime }1^{\prime }}^{i}-N_{3^{\prime }3^{\prime }}^{i})];\\ \chi_{s}=-D^{-1}\gamma^{2}M_{0}^{2}\sum_{i}N_{1^{\prime }2^{\prime }}^{i};\\ \chi_{a}=D^{-1}\gamma M_{0}\omega ;\\ D=\omega_{0}^{2}-\omega^{2};\\ \omega_{0}^{2}=\gamma^{2}[H_{03^{\prime }}^{}+\sum_{i}(N_{1^{\prime }1^{\prime }}^{i}-N_{3^{\prime }3^{\prime }}^{i})M_{0}][H_{03^{\prime }}^{}+\\ +\sum (N_{2^{\prime }2^{\prime }}^{i}-N_{3^{\prime }3^{\prime }}^{i})M_{0}]-(\sum_{i}N_{1^{\prime }2^{\prime }}^{i}M_{0}{)}^{2}, \end{array}$$

where γ, ω, and $N_{k^{\prime }n^{\prime }}^{i}$ are the magneto-mechanical ratio, angular frequency, and demagnetization coefficients corresponding to all types of magnetic anisotropy with $1^{\prime },2^{\prime },3^{\prime }$ denoting a frame of axis with $3^{\prime }$-axis directed parallel to the equilibrium magnetization. Equation (13) comprises magnetic form anisotropy, magnetic crystallographic anisotropy, and electric field induced magnetic anisotropy. Demagnetization coefficients arising from the magnetic crystalline anisotropy *N^a^_ik_* and magnetic anisotropy *N^E^_ik_* induced by dc electric field are specified by following equations:

(14) Na11− Na33=[2 (β431′+ β432′+ β433′) − 6(β211′β231′+ β212′β232′+ β213′β233′)]Ha/M0;Na22− Na33=[2(β431′+ β432′+ β433′) − 6(β221′β231′+ β222′β232′+ β223′β233′)]Ha/M0;NknE=2bijknTijmβkk′βnn′,

In Equation (14), *b*_1111_ = *b*_2222_ = *b*_3333_ = 3*λ*_100_*/*(2*M*_0_^2^*), b*_1212_ = *b*_1313_ = *b*_2323_ = 3*λ*_111_*/M*_0_^2^, *λ*_100_ and *λ*_111_ are magnetostriction coefficients, and *^m^T_ij_* is the electric field induced stress component. *β* is direction cosine matrix corresponding to axes (1,2,3) with respect to the axes (1′,2′,3′).

Solving the linearized equation of motion for magnetization with dissipative term *iωa (M*_0_ *× m)/M*_0_ (*a* is the dissipation parameter) enables one to get the expressions for the magnetic susceptibility component *χ*_1_ = *χ*’ + *i χ*″ in the form:

(15) $$\chi^{\prime }=\chi_{0}\frac{\omega_{0}^{2}\left(\omega_{0}^{2}-\omega^{2}+2\alpha^{2}\omega^{2}\right)}{{\left(\omega_{0}^{2}-\omega^{2}\right)}^{2}+4\alpha^{2}\omega_{0}^{2}\omega^{2}},\;\chi^{\prime \prime }=\chi_{0}\frac{\alpha \omega \omega_{0}\left(\omega_{0}^{2}+\omega^{2}\right)}{{\left(\omega_{0}^{2}-\omega^{2}\right)}^{2}+4\alpha^{2}\omega_{0}^{2}\omega^{2}},\;\chi_{0}=\gamma \frac{M_{0}}{\omega_{0}}.$$

To find the electric field induced stress, we employ the elasticity equation for piezoelectric layer:

*^p^S*_1_ = *^p^d*_31_*^p^E*_3_ + *^p^s*_11_*^p^T*_1_ (16)

where *^p^E*_3_ is the internal electrical field; *^p^S*_1_, *^p^d*_31_, and *^p^s*_11_ are the deformation, piezoelectric constant, and elastic flexibility of the piezoelectric layer, correspondingly. 

As an example, we suppose that the piezoelectric layer is polarized along the [111] axis of ferrite phase. Elasticity equation for ferrite layer is as follows:

*^m^S*_1_ = *^m^s*_11_*^m^T*_1_ (17)

Here *^m^T_i_* and *^m^S_k_*, are the stress and deformation components of the ferrite, correspondingly.

The layers are assumed rigidly connected and the ferrite layer prevents piezoelectric layer’s free contraction when dc external electric field is applied. The forces produce bending moments on each layer due to asymmetry of the structure. To take account of the flexure, the axial deformations of layers have to be dependent on coordinate *z_i_* [14]:

*^m^S*_1_ = *^m^S*_10_ + *z_m_*/*R*;  *^p^S*_1_ = *^p^S*_10_ + *z_p_*/*R*; (18)

where *^m^S*_10_ and *^p^S*_10_ are the deformations along *x* axis corresponding to layer’s middle plane and *R_1_* is the bend radius, *z_m_* and *z_p_* are counted off from the middle plane of layers.

From geometric consideration, one can see that *^m^S*_10_ *− ^p^S*_10_ = *h_m_/R* where *h_m_* = (*^m^t* + *^p^t)/2* with *^m^t* and *^p^t* being the thicknesses of ferrite and piezoelectric phases.

For the sample in equilibrium state, the resultant force and moments should vanish:

*F_m_*_1_ + *F_p_*_1_ = 0, *F_m_*_1_*h_m_* = *M_m_*_1_ + *M*_p1_ (19)

where Fi1=∫−ti/2it/2iT1dzi and Mi1=∫−ti/2it/2ziiT1dzi are the transverse force and bending moment per unit width.

For solving Equation (19) for *^m^S*_10_ and *R*, the stress components should be expressed from Equations (16) and (17). Substituting the centroidal strains and radius of curvature into the Equation (19) enables finding the axial stress *^m^T*_1_. Note that *^m^T*_1_ depends on *z* due to flexural deformations. Taking into account Equations (17) and (18) and found *^m^T*_1_, Equation (19) results in magnetic susceptibility components that vary with *z*.

Bending the sample have two implications: (i) it reduces the stress of ferrite layer and thus ME effect, (ii) the actual manuscript provides evidence that flexural deformations lead to the electric field induced broadening of FMR line. The FMR line broadening can be estimated as the difference between two peaks in magnetic field dependence of *χ*″.

We assume magnetic anisotropy induced by dc electric field small compared to other types of anisotropy. The FMR line shift can be expressed for this case in the linear approximation [13]:

(20) $$\delta H_{E}=-\frac{M_{0}}{Q_{1}}\left[Q_{2}\left(N_{11}^{E}-N_{33}^{E}\right)+Q_{3}\left(N_{22}^{E}-N_{33}^{E}\right)-Q_{4}N_{12}^{E}\right]\;,$$

where:

$$\begin{array}{l} Q_{1}=2H_{3}+M_{0}\sum_{i\neq E}\left[\left(N_{11}^{i}-N_{33}^{i}\right)+\left(N_{22}^{i}-N_{33}^{i}\right)\right]\;;\\ Q_{2}=\left[H_{3}+M_{0}\sum_{i\neq E}\left(N_{22}^{i}-N_{33}^{i}\right)\right]\;;\\ Q_{3}=\left[H_{3}+M_{0}\sum_{i\neq E}\left(N_{11}^{i}-N_{33}^{i}\right)\right]\;;\\ Q_{4}=2M_{0}\sum_{i\neq E}N_{12}^{i}\;. \end{array}$$

**Figure 16:**
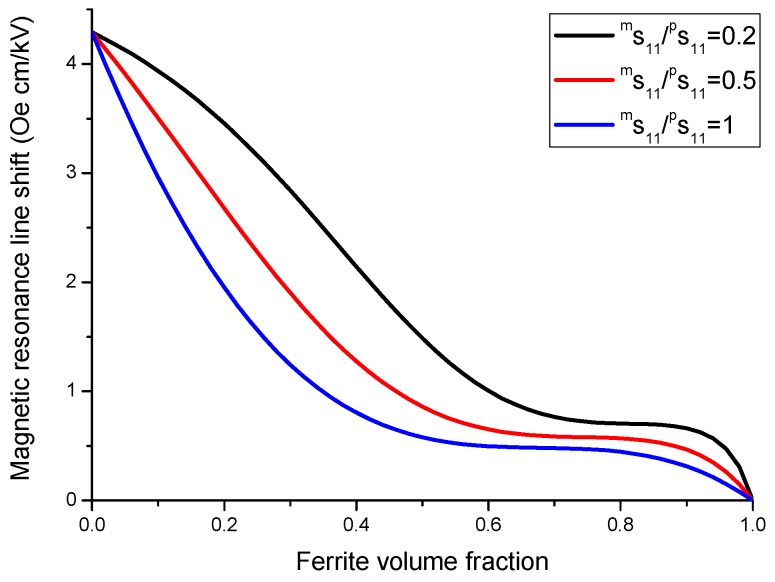
Variation of the shift of FMR field with volume fraction of ferrite phase at E = 1 kV/cm for ferrite-piezoelectric laminate for $\left|\frac{\lambda_{111}}{M_{s}}\right|=0.16\times 10^{-8}\;Oe^{-1}$ [27].

**Figure 17:**
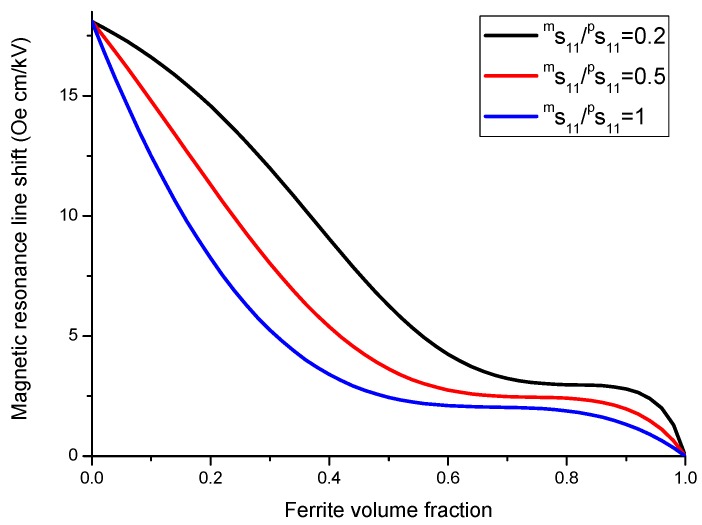
Variation of the shift of FMR field with volume fraction of ferrite phase at E = 1 kV/cm for ferrite-piezoelectric laminate for $\left|\frac{\lambda_{111}}{M_{s}}\right|=0.68\times 10^{-8}\;Oe^{-1}$ [27].

**Figure 18:**
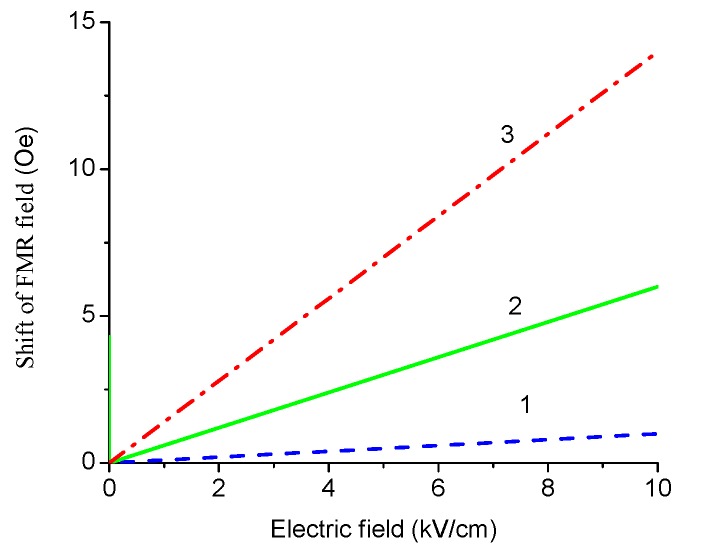
Variation of the shift of FMR line with dc electric field at 9.3 GHz for the laminates of YIG and PZT (**1**), NFO and PZT (**2**), and LFO and PZT (**3**) with equal thicknesses of ferrite and piezoelectric layers.

As an example, we apply our model to a specific direction of bias field *H* parallel [111] axis. Variation of the shift of FMR field with volume fraction of ferrite phase is given in Figure 16 and Figure 17. Dependence of FMR field displacement on applied electric field is shown in Figure 18.

**Figure 19:**
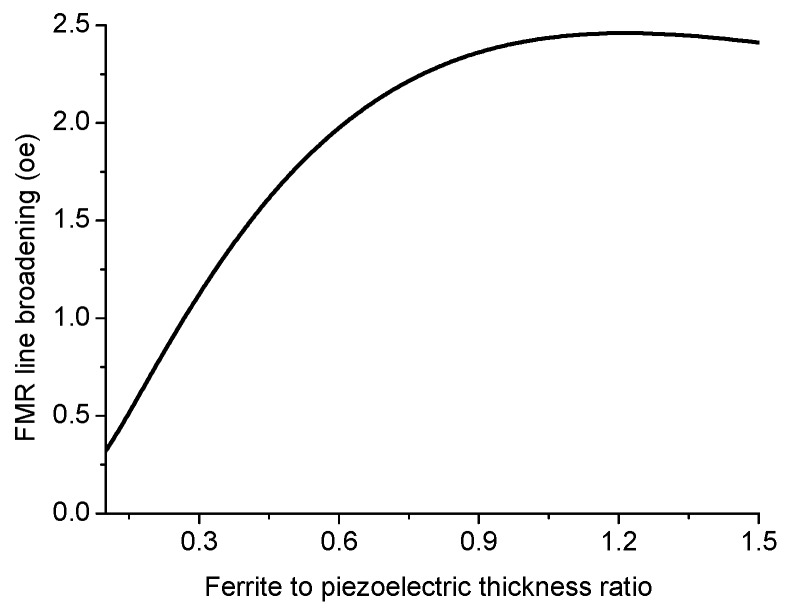
Variation of FMR line broadening with ferrite to piezoelectric thickness ratio at E = 6 kV/cm for ferrite-piezoelectric bilayer with $\left|\frac{\lambda_{111}}{M_{s}}\right|=0.16\times 10^{-8}\;Oe^{-1}$.

**Figure 20:**
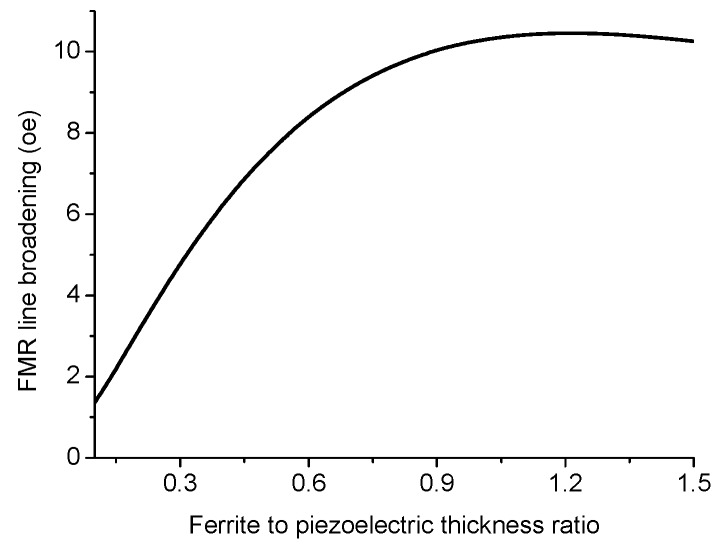
Variation of FMR line broadening with ferrite to piezoelectric thickness ratio at E = 6 kV/cm for ferrite-piezoelectric bilayer with $\left|\frac{\lambda_{111}}{M_{s}}\right|=0.68\times 10^{-8}\;Oe^{-1}$.

Variation of FMR line broadening with ferrite to piezoelectric thickness ratio is given in Figure 19 and Figure 20.

## 6. Discussion

Figure 1 and Figure 2 present the plots of ME voltage coefficients vs. piezoelectric volume fraction in the low-frequency region for transverse fields’ orientation for symmetric structures. It should be noted that under a substitution of *q*_11_ in Equation (2) with *q*_31_, one can derive the longitudinal ME voltage coefficient. Similarly, the in-plane longitudinal ME coefficient is derivable from Equation (2) by correspondent replacement of *d*_31_ for *d*_33_. The strongest ME effect is predicted for the case when dc and ac magnetic and electric fields are in the sample plane.

Plot of ME voltage coefficient vs. volume fraction of piezoelectric phase for bilayers is a double-peaked curve (Figure 3 and Figure 4). That is accounted for by the contribution of bending strain to the total one. When we neglect the flexural deformation, the ME voltage coefficient is a single-humped function of volume fractions. When the two types of strains superimpose on one another, one can see damping of *α_E,_*_31_ in the centre and the plot of ME voltage coefficient vs. volume fraction of piezoelectric phase becomes a double-peaked curve. However, variation of ME voltage coefficient with volume fraction of piezoelectric phase is a single-peaked curve for laminate of magnetostrictive layer and stepped piezoelectric as in Figure 5, Figure 6 and Figure 7. This is due to the fact that compositionally stepped structure produces a change in contribution of flexural strain to the total one. In addition, the maximal ME effect is obtained at higher piezoelectric volume fraction compared to bilayer of homogeneous components. 

The maximal ME voltage coefficient in the bending mode region is obtained for lower piezoelectric volume fraction compared longitudinal modes that corresponds to maximum flexural deformation (Figure 8 and Figure 9). However, there is a rather weak nonlinear dependence of resonance frequency on volume fraction of piezoelectric phase (Figure 10 and Figure 11). It is related to the fact that the cylindrical stiffness is curvilinear function of piezoelectric volume fraction. Note that the transverse ME coefficient is larger than longitudinal one because of a decrease in internal magnetic field due to demagnetizing effect for the longitudinal fields’ orientation. The bending resonance frequency and peak ME coefficient depend on conditions of end restraint for a bilayer. In this manuscript, we investigated the laminates with single-sided support because the strong ME coupling is obtained at lowest bending resonance frequency.

ME coupling in structures of stepped piezoelectric and magnetostrictive layer is predicted to be two times stronger in the low-frequency region (Figure 5 and Figure 6) and 1.5 times stronger in the bending mode region than for the traditional piezoelectric/magnetostrictive bilayer (Figure 12 and Figure 13). The obtained increase in ME coupling strength is attributed to the flexure produced stress redistribution in piezoelectric layers that gives rise to an enhancement of output voltage at specified volume fractions of piezoelectric phase.

The resonance enhancement of ME coupling in the longitudinal mode region is determined by Equation (6). ME coupling in the EMR region is two orders stronger than that in the low-frequency region. The plot of *α_E_*_,13_ vs. volume fraction of piezoelectric phase has a maximum at specific volume fraction. Note that Equations (11) and (12) for EMR frequency and maximal ME voltage coefficient at EMR are directly applicable both to bilayer and trilayer laminates (Figure 14 and Figure 15). The strongest ME effect at EMR can be obtained for the in-plane longitudinal magnetic and electric fields’ orientation as is the case of the low-frequency ME effect. From Equation (12), it can be inferred that the ME voltage coefficient divided by Q is a function of piezoelectric phase volume fraction and this function is analogous to that for low frequency region.

The results in Figure 16, Figure 17 and Figure 18 show that for obtaining the maximal ME effect in FMR region: (i) the volume fraction of the ferrite phase should be as low as possible; (ii) piezoelectric coefficient of piezoelectric layer should be as high as possible; (iii) ratio of magnetostriction constant to saturation magnetization should be as high as possible [19]. It should be noted that a dc electric field applied to piezoelectric component gives rise to a uniaxial thickness-dependent magnetic anisotropy that results in FMR line broadening. Microwave resonators with electrically tuned resonance frequency and bandwidth are of importance for signal processing devices such as controlled filters.

To apply the nomographs method for evaluation of ME coefficients, one should use the relevant material parameters of laminate components. The initial parameters for some of piezoelectric and magnetostrictive materials are provided in Table 1.

As an example of ME laminate would be the bilayer of Ni and PZT with equal content of magnetostrictive metal piezoelectric ceramic. Using the data from Table 1, we obtain *^p^s*_11_ = 15.3 × 10^−12^ m^2^/N, *^p^d*_31_ = −175 × 10^−12^ m/V, *^p^ε*_33_/*ε*_0_ = 1750, *^m^s*_11_ = 20 × 10^−12^ m^2^/N, *^m^q*_11_ = −4140 × 10^−12^ m/A. Figure 4 shows that the static ME voltage coefficient equals *α_E_*_,31_ = 190 mV/(cm·Oe). In addition, Figure 2 and Figure 5 provide the maximal ME voltage coefficient *α_E_*_,31_ = 20 V/(cm·Oe) for flexural mode frequency and *α_E_*_,31_ = 70 V/(cm·Oe) for longitudinal mode frequency. We used Q-value to be equal to 100 for both modes.

## 7. Conclusions

ME laminates can be used for obtaining ME coupling which is considerably stronger compared to that for single crystals. The development provides opportunities for ME laminates to be applied in functional electronic devices, such as sensors, energy harvesters, microwave resonators and filters etc. We presented here a new rapid test method for ME laminates based on the graphs of ME voltage coefficients against composite parameters. This method can be used when rough estimates are relevant and helpful. To plot the ME parameters versus initial material parameters and component volume fractions, we modelled the magnetic field induced ME effect for several operational modes and laminate composites of most importance to application in electronic devices. In particular, we considered longitudinal and bending modes, laminates of homogeneous and compositionally stepped piezoelectrics and magnetostrictive materials.

Another area of application of obtained results is estimating the volume fractions of initial components to obtain the highest ME coefficients. Additionally, one can benefit directly from a graph to validate calculation data that results from another computational technique even though the accuracy of graphs is generally poorer. 

In spite of the fact that some devices based on multiferroic layered structures have been proposed, much work should be done for real applications. Recently, Metglas ribbons were used as the magnetostrictive component in multiferroic composites. Metglas has an ultrahigh permeability and as a result the high piezomagnetic coefficient at weak bias magnetic field. An internal magnetic field can be produced by using the compositionally graded structure [16]. Accordingly, a Metglas based multiferroic structure can be considered as a candidate for incorporation into ME composite to get a largely enhanced ME coupling at zero bias. Clearly, magnetostrictive-piezoelectric composites including nanocomposites with zero-bias ME effect are important for functional device development. Not that the affect of polymer binders on the ME response should be studied.

## Figures and Tables

**Table 1:** Piezoelectric, magnetostrictive, piezomagnetic, and elastic parameters of materials for manufacturing of ME laminates.

Material	*s*_11_ (10^−12^ m^2^/N)	*s*_12_ (10^−12^ m^2^/N)	*q*_33_ (10^−12^ m/A)	*q*_31_ (10^−12^ m/A)	*d* (10^−12^ m/V)	*λ*_100_ (10^−6^)	*ε*_33_/*ε*_0_
PZT	15.3	−5	-	-	*d*_31_=−175	-	1750
BTO	7.3	−3.2	-	-	*d*_31_=−78	-	1345
PMN-PT	23	−8.3	-	-	−600		5000
Langasite	8.8	−4.3	-	-	*d*_14_ = −3.65		50
Langatite	9.8	−3.8	-	-	*d*_14_ = −2.81		77
Quartz	12.8	−1.8	-	-	*d*_14_ = −0.67		4.68
LiNbO_3_	4.9	1.8			*d*_15_ = 69		84.6
AIN	3.1	0.8			*d*_31_ = −2.8		8.8
YIG	6.5	−2.4			-	1.4	10
NFO	6.5	−2.4	−680	125	-	23	10
LFO	35	−12				46	10
Ni	20	−7	−4140	1200			
Terfenol-D	33.3	−10	15,707	4730	-		
Metglas	10	−3.2	14,000	−3000	-	-	
